# Gutta Percha Gums

**Published:** 1856-06

**Authors:** 


### GUTTA PERCHA GUMS.

It is but a few months since Dr. Slayton introduced his colored specimens of gutta percha, though at present nearly every well informed dentist throughout the Union is acquainted with it. Previous to his mixture, there were several methods of making and applying this material to supply deficiencies in the mouth. For several years white gutta percha has been used in England, Scotland and France, and its indestructibility well attested; but its use was more particularly confined to lining metalic plates for the relief of pain where the patient's gums were in an unhealthy condition; and it was but seldom used for imitation of natural gums; but when so applied, it was painted the proper color, and warmed over a spirit lamp, in order for the coloring matter to be partially absorbed in its surface, and thereby prevented from fading. The art of coloring and applying it was done according to the various ideas that any dentist might chance to hit upon; but the great difficulty under which they all labored was, bungling or clumsy work and poor colors. These difficulties now seem to be entirely overcome, by applying it only for gums on gold plate work, and by coloring and preparing it perfectly pure. Slayton's mixture was, no doubt, far superior to what had formerly been in use; but its texture rendered it unfit for any thing but temporary work, and the latest manufactured, is scarcely fit for that; but with what result the various experience of dentists has proved, one can scarcely form an estimate of its utility, or of its countless failings in doing any service whatever. The opposite views that dentists entertain about it, are not owing so much to the difference in their talents, as to the experience they have had with it; and this experience too, depends less upon the position of the dentist, the city, or street, that he may chance to inhabit, than it does on his disposition to take hold of, and his perseverance to carry out what promises good to his profession. While there are many of our "first dentists" who denounce everything that was unknown to their preceptors, and whose obstinacy induces them to condemn every article they are unacquainted with, there are, also, many more who eagerly and rashly grasp after everything new. Between these two classes of dentists, there is a perpetual warfare; and in childish battles they are continually branding each other, with the petty names of "old fogy" and "humbug." The old fogies are generally aged men and old in the profession, with a practice sufficient to live at ease, without this jealousy toward the others; but the humbugs are later in the profession, and generally read and reflect extensively, in hopes of discovering some new item that will insure a more profitable practice. But so eager are they in the pursuit, that they often take for evidence and utility, that which maturer reflection discoveres to be defective; and each of their failures confirms the old fogy that he knew it would be so, and saves him a private chuckle for knowing too much to enter the speculation. From either of these, it is evident we can gain but little reliable information; for though they may be well informed, yet their extreme notions and prejudices render their views defective.

From these sources, gutta percha has been lauded by the one, and denounced by the other; although, however, he who has tried it, possesses information of which the other is entirely destitute. These humbugs, in their antagonists eyes, comprise all dentists who have tried crystal gold, continuous gum work, local anasthesia, and gutta percha; but in their opinions, the old fogies comprise all dentists from barber tooth drawers, to the Bond street stiffness, that bellows out against everything new. The speculative class, however, need another division, such, for instance, as approach nearly to the old fogy order, and who generally rest on their oars until new things are tested, and then come in time to reap the profits with out expense to themselves. Among this latter class are some of our most scientific dentists; and although they deserve less credit for their information than those air-castle experimentalists, who devote so much of their time to so little profit, yet they are the standard where our confidence inclines to rest. This class of men are always prepared to investigate when no money or time is required; and trusting so much upon the experience of their more enthusiastic brethren, they are better able to collect that which is useful, and to reject whatever has proved a failure. Acting upon this ground, they have dipped cautiously into gutta percha, knowing from hear-say evidence, that it was a gum possessing indestructible qualities, and that with proper preparation it might be introduced in the mouth, instead of gold and porcelain work. But their trials, throughout the whole country, have met with many obstacles, and also with many probabilities of usefulness. The quality of the gutta percha was almost entirely overlooked; and the filthiest kind of an article, with a tinge of brick dust paint, as unlife-like as sun dried sole leather, was quickly crowded into every dental depot, at an "enormous cheap" price on account of its great secresy. From Long Island to St. Louis, full sets could be put in for twenty dollars, warranted for five years, "unless the patient used tobacco!" But this would not do. Many a good dentist "loathed returning faces," and soon found many a "bad taste" and "faggy fringes" coming back again. Here, the too cautious ones backed out, and the cheap ones, and such as have chanced to get good gutta percha, or who are fortunate enough to know how to prepare it, are the only ones who continue to use it. And here, too, arises another consideration that has always followed "dental discoveries;" that is, to get a cheap article, so cheap that poor people could afford to have teeth put in! Cheap gutta percha seemed to be the unanimous wish; and no doubt if it could be prepared for fifty cents a pound, dentists would want it mixed with something else, in order to make it still cheaper. Of some of the gutta percha now in our various markets, it is scarcely possible to believe what substances some of these genii have introduced, in order to give it its "wonderful color." In almost the whole of it, can be detected its native impurity, sap, and even pieces of bark, chips and such substances as exist in the common sole leather article of commerce.

With such impurities in it, it is not at all strange that decomposition, bad taste, smell, etc., would result from its introduction in the mouth, and not strange either that many cautious dentists should discontinue its use, without even attempting to discover wherein the difficulties had arisen. But the above impurities were not all: in its preparation, some of these cheap manufacturers had introduced many mineral and vegetable poisons, in the form of fine powder. These articles have been kneaded into it, to bring up to its wonderful beauty, without the apprehension of these newly made chemists, that powdered substances injured the toughness of the gum, and rendered it liable to "wear raggedly." The amount of these powders introduced can scarcely be believed. In analyzing five ounces of the gum colored, about fifteen per cent of white zinc was detected; and in a small quantity of the "White Stopping," over forty per cent. was found. Indeed it seems as if some of these wonderful discoverers have merely kneaded white zinc into crude gutta percha, until nearly white, and then, by the addition of Carmine and Canada Balsam, given it the dead hue of which it is characteristic. In one of the best articles now in market, was found a small quantity of one of the preparations of gold, intended for coloring porcelain enamel. What that individuals notion was, of coloring gums, can only be conceived by imagining his pockets overrun with the metal, for certainly he must have known that without being fused, it could impart no more brilliancy than many other minerals. All of these colors are, however, superior to the European method of painting and varnishing; but the texture of the gum in nearly all that is in use has been injured in preparing it, and its natural gloss has been destroyed. There are, however, a few persons who have been fortunate enough to ascertain the manner of purifying and coloring it without injuring its toughness, hardness, and gloss, who have not been annoyed with the difficulties under which the mass have labored; and these difficulties, too have been quite sufficient to discourage all persons belonging to either the "old fogy" or the "humbug" order, whose whimsical views rested mostly upon the opinions of other persons.

With such gutta percha as the markets are now glutted, it is not surprising that nearly every dentist should pronounce a "temporary" sentence as its eternal doom; and among their reasons for such a decision are, its disagreeable smell, its deadness in color, its liability to separate from the teeth, and its want of hardness and toughness. They admit its power to resist acids and alkalies, yet fail to detect its impurities, wood and sap, which cause it to smell and decompose. They admit it has a natural gloss, but forget that their coloring powders destroy it. They know its natural adhesiveness, but fail to discover why it leaves the teeth. They know that crude gutta percha is almost as hard as horn, yet judge that it cannot be prepared hard enough for artificial gums! More than this, too, every well read dentist knows that in England, gutta percha has been used as a base for plate-work for many years; and, with the exception of color, it has been practically tested sufficient to prove its durability for many dental purposes. But that it ever can be so prepared as to answer for plates instead of gold seems highly improbable, although its indestructible qualities may be a safe guarantee for its use in making permament gums on gold plate work. Though for such a purpose, it is necessary that it should be perfectly pure, and that its colors be equally life-like to porcelain gums; but there are many difficulties to be overcome in accomplishing these points. There is no guarantee in buying a pure article, and few dentists know how to prepare it so, besides, its colors must be granulated in order to retain its gloss; and this cannot be done except the dentist prepares his shades and tints after he has finished using his spatula upon it. The difficulty, however, of getting it to adhere to the plate and teeth, is overcome by the band work. This band, when quite warm, having been drawn round the gums just above the teeth, is so imbedded in the gutta percha, that it can be afterwards entirely covered over with it, and as each end of the band is riveted to the main plate, the gutta percha rests so snugly between the two, that the whole job is quite as durable as continuous gum work. And when "Allen's Teeth" are used, and the backings joined so closely as to make a tight, smooth job on the lingual side, the work has many advantages that no other has, such as lightness, living expression, ease in putting it up, and capacity to fill up inequalities in the alveolar ridge.

Under such precautions, gutta percha promises more advantages than any of the discoveries or applications that have ever been introduced; the wear and tear of gums on plate work, being so trifling that from the apparent hardness of pure gutta percha, but little is to be feared that it will not answer for nearly all permanent work. One of the chief objections is, however, in the persons who use it. It is so easily colored by kneading ingredients into it, that "bogus" work would be likely to supersede that which *can be manufactured.* Even Slayton's make was at first *partially* purified; but at present it is so impure as to be quite unlike the original; and the manufacturers of it *acknowledge that the competition in price prevented them from making it after Slayton's origi-*

*nal formula.* If that statement be true, and if one hundred per cent be profitable, they betray a good deal of ignorance in manufacturing, even the present impure article. Trusting that dentists will try to apply it on gold plate work, and that they may easily perceive the nice and cleanly advantages it has over single gum teeth, the matter shall be left here for their private consideration.

